# Increased Serum Interleukin-6 and Tumor Necrosis Factor Alpha Levels in Fabry Disease: Correlation with Disease Burden

**DOI:** 10.6061/clinics/2021/e2643

**Published:** 2021-07-08

**Authors:** Nilton Salles Rosa, Judith Campos de Barros Bento, Valéria de Falco Caparbo, Rosa Maria Rodrigues Pereira

**Affiliations:** Divisao de Reumatologia, Faculdade de Medicina FMUSP, Universidade de Sao Paulo, Sao Paulo, SP, BR.

**Keywords:** Fabry Disease, *GLA* Gene, Inflammation, Mainz Severity Score Index, Cytokine

## Abstract

**OBJECTIVES:**

Fabry disease (FD) is an X-linked lysosomal disease caused by variants of the *GLA* gene; the formation of defective alpha-galactosidase A contributes to the accumulation of substrates in several organs. Chronic inflammation is thought to contribute to organ damage in FD patients.

**METHODS:**

In total, 36 classic FD patients (15 men/21 women) and 25 healthy controls (20 men/8 women) were assessed. The Mainz Severity Score Index (MSSI) was established after conducting interviews with the patients and chart review. Serum IL-6, IL-1β, and TNF-α levels were evaluated in both groups.

**RESULTS:**

The mean age (years) for FD patients was 43.1±15.4 and that for the controls was 47.4±12.2 (*p*>0.05). Twenty-two patients (59.5%) were treated with enzyme replacement therapy (ERT). Serum IL-6 and TNF-α levels were significantly higher in FD patients than in the controls. Patients treated with ERT had higher serum IL-6 and TNF-α levels than those not treated with ERT. There was no difference in the serum IL-1β levels between patients treated with ERT and those who were not. The MSSI scores in the patients were correlated with serum levels of IL-6 (r=0.60, *p*<0.001) and TNF-α (r=0.45, *p*<0.001).

**CONCLUSION:**

FD was associated with elevated serum levels of IL-6 and TNF-α in this cohort. The FD patients treated with ERT, particularly, women, exhibited higher levels of serum IL-6 and TNF-α than those not treated with ERT; the serum IL-6 and TNF-α levels were correlated with the MSSI scores reflecting greater disease burden.

## INTRODUCTION

Fabry disease (FD) is an X-linked lysosomal disease caused by pathogenic variants of the *GLA* gene, which encode a defective alpha-galactosidase A enzyme, contributing to substrate accumulation in several organs, with varying degrees of severity and subsequent loss of organ function (1,2).

The natural role of lysosomes includes not only substrate degradation, but also the exocytosis of proteins and vesicles, plasma membrane repair, remodeling and growth, inter-organelle and inter-cellular signaling, metabolic sensing, lipid metabolism, and response to cell injury (3). Moreover, as regulators of the immune system, lysosomes are also active players in antigenic presentation and processing, secretion of perforins, phagocytosis, and release of pro-inflammatory mediators (4). Progressive Gb3 accumulation inside lysosomes is not sufficient to explain all aspects of FD pathology. Nevertheless, it is the starting point for a cascade of cellular events that ultimately disrupts the normal functioning of cells (1,4-6).

After the initial trigger, it is thought that energetic metabolism becomes impaired, oxidative stress attains a pro-oxidative status, and autophagosomes fail to mature efficiently. Eventually, dysfunction of the ionic membrane channels ensues. In due course, injury of small vessels results in tissue ischemia, cell death, and development of irreversible tissue fibrosis, most prominently affecting the heart and kidneys (1,7-12).

In this context, chronic inflammation is thought to contribute to organ damage in patients with FD. Previous reports have evaluated the plasma levels of pro-inflammatory cytokines (7,13-16), peripheral blood mononuclear cell (PBMC) levels of pro-inflammatory cytokines (16,17), and functional polymorphisms of pro-inflammatory cytokine-encoding genes in patients with FD (18).

This study aimed to evaluate the levels of serum inflammatory cytokines in patients with FD, compared to those in healthy controls, and to ascertain their correlations with the Mainz Severity Score Index (MSSI).

## METHODS

This is a sub-analysis of the laboratory findings from the protocol “Assessment of Parameters of Bone Metabolism in patients with Fabry Disease,” which was undertaken at the Rheumatology Division, Faculdade de Medicina, Universidade de São Paulo, São Paulo, Brazil. This work was approved by the Faculdade de Medicina da Universidade de São Paulo Ethics Review Board (number 1.464.841) on March 24, 2016. Written informed consent was obtained from all patients. All procedures were in accordance with the ethical standards of the responsible committee on human experimentation and with the Helsinki Declaration of 1975, as revised in 1983.

Thirty-six genotype-confirmed classic FD patients - 15 men and 21 women (variants: C142R, A156D, L180F, R227X, W262X, G271A, P293S, and Y264SX) - and 25 age-matched healthy controls (20 men and 8 women) were assessed. The MSSI (19) was established after interviews with the patients and chart review. Because this is a transverse study, it is not possible to have a trend of MSSI values. Data regarding the age at symptom onset, age at diagnosis, and age at treatment initiation have been previously published (20). In summary, the median age at symptom onset was 12.0 years (range, 5-55 years) and 16.5 years (range, 9-66 years) for men and women, respectively. The mean age at diagnosis was 35.5 years for men (range, 8-55 years) and 35.9 years for women (range, 4-71 years). The median time to diagnosis after symptom onset was 20.5 years (range, 1-42 years) for men and 14.0 years (range, 0-52 years) for women (20).

Serum interleukin (IL)-6, IL-1β, and tumor necrosis factor (TNF)-α levels were evaluated using the HBNMAG-51K-13 kit. The human Milliplex^®^ assay was performed using the Luminex^®^ technology kit (Millipore Corporation, Billerica, MA, USA), which is a multi-analyte panel used for bone metabolism research and measures the IL-6, IL-1β, and TNF-α levels.

### Statistical analysis

Results are presented as the mean and standard deviation for continuous variables and percentages for categorical variables. Correlations between continuous variables were measured using Pearson’s correlation coefficient. Statistical significance was set at *p*<0.05.

## RESULTS

The mean age (years) of the FD patients was 43.1±15.4 and that of the control subjects was 47.4±12.2 (*p*>0.05). Twenty-two patients (59.5%) were treated with enzyme replacement therapy (ERT) and no patients were treated with chaperone therapy during the assessment period.

**Figure 1:**
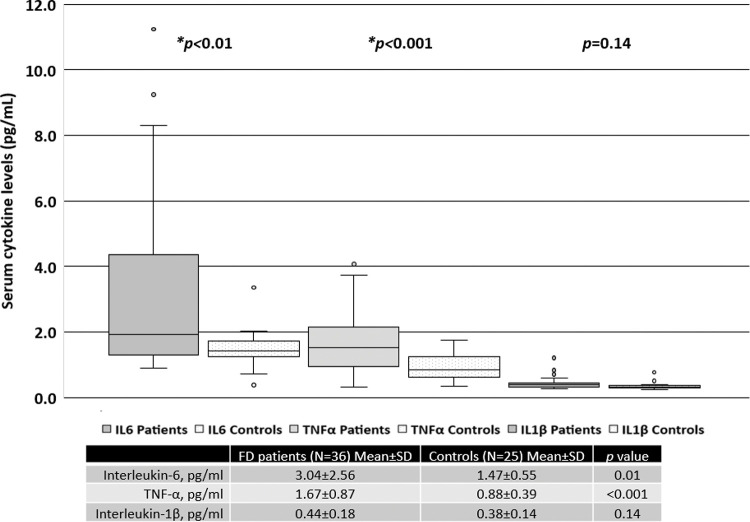
Serum cytokine levels in FD patients *versus* those in the control subjects.

When all patients were included in the analysis, the serum levels of IL-6 and TNF-α were significantly higher than those in the healthy controls, as shown in Figure 1. There was no difference in the levels of IL-1β between FD patients and the controls.

**Figure 2:**
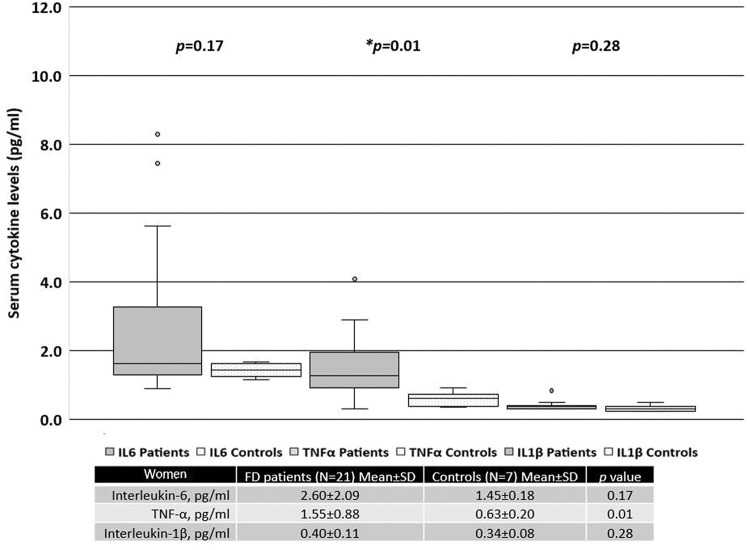
Serum cytokine levels in women with FD *versus* those in the control subjects.

**Figure 3:**
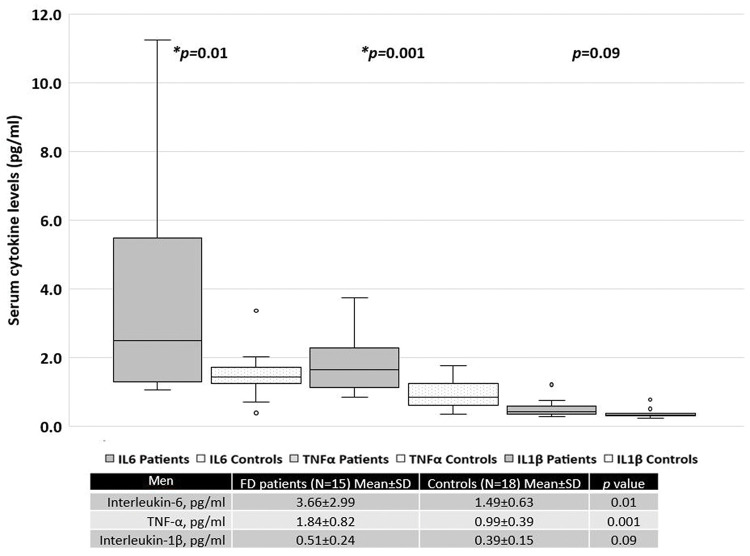
Serum cytokine levels in men with FD *versus* those in the control subjects.

When patients were separated based on sex, women with FD had significantly higher serum levels of TNF-α than the healthy female controls (Figure 2). In contrast, men with FD had significantly higher serum levels of IL-6 and TNF-α than the healthy male controls (Figure 3).

**Figure 4:**
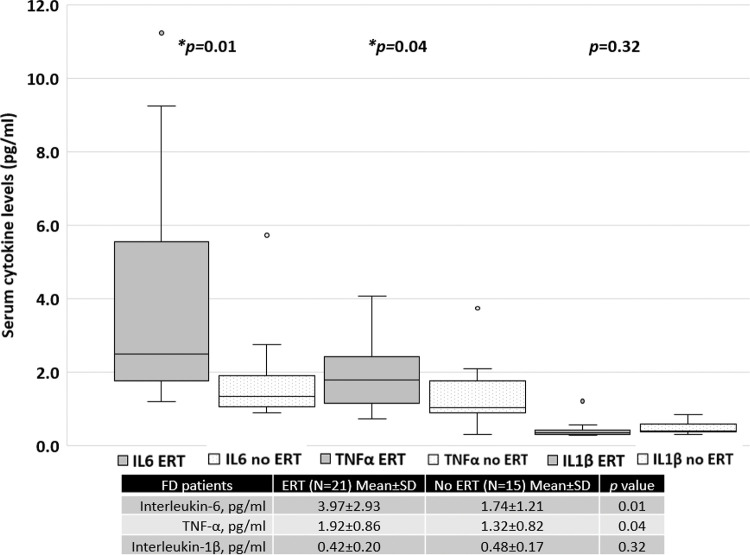
Serum cytokine levels in FD patients treated with ERT *versus* those not treated with ERT.

Patients treated with ERT exhibited higher serum IL-6 and TNF-α levels than those not treated with ERT, as shown in Figure 4. No difference was found between the serum IL-1β levels of patients treated with ERT and those that were not. Cytokine levels in patients treated with and without ERT separated based on sex are available in the Appendix.

The MSSI separates patients into three categories based on severity: low, moderate, or severe. Six patients showed severe FD (1 women/5 men); 14 patients showed moderate FD (8 women/6 men), and 17 patients showed low-severity FD (12 women/5 men).

The mean MSSI score in men with FD who were treated with ERT was not different from that of patients not treated with ERT (29.0±11.1 *versus* 26.7±14.9, *p*=0.78). In contrast, in women with FD, the mean score was 25.8±8.2 for those treated with ERT and 10.3±7.1 for those not treated with ERT (*p*=0.0003).

**Figure 5:**
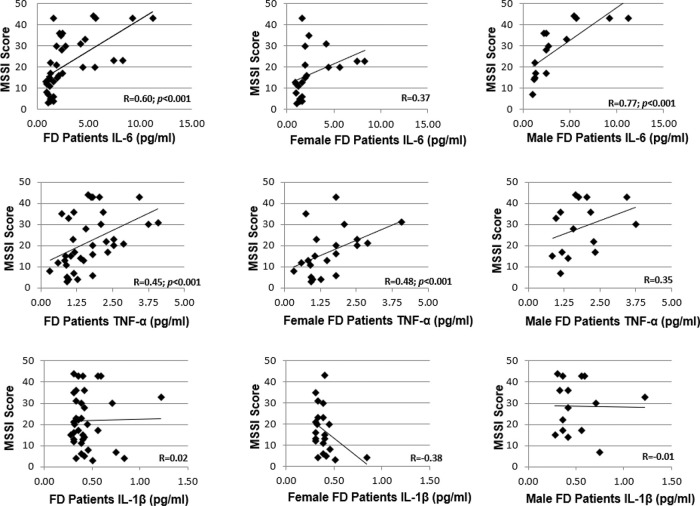
Correlations between MSSI scores and serum cytokine levels in men and women with FD.

In all patients, the MSSI score correlated with the serum levels of IL-6 (r=0.60, *p*<0.001) and TNF-α levels (r=0.45, *p*<0.001), as shown in Figure 5. However, the serum IL-1β levels did not correlate with the severity score (r=0.02). When separated based on sex, a correlation with the MSSI score was observed only for TNF-α levels (r=0.48, *p*<0.001) in women with FD and only for IL-6 levels (r=0.77, *p*<0.001) in men with FD.

## DISCUSSION

The evidence presented in this study reinforces the fact that FD patients have elevated serum levels of pro-inflammatory cytokines. The disease burden, as estimated by the MSSI score, is clearly related to higher levels of IL-6 and TNF-α.

The pro-inflammatory state has previously been described in FD. There is an accumulation of Gb3 in leukocytes and subsequent changes in the subpopulations and expression of surface molecules (4,10,12,21). Invariant natural killer T cells (iNKT) recognize the glycosphingolipid Gb3 as an antigen presented by antigen-presenting cells and promote the release of inflammatory cytokines (22,23). In addition, Gb3 may work as an activator of Toll-like receptor 4 (TLR4) and contribute to the deregulation of the activity of both iNKT cells and dendritic cells (4,16,23).

Lysosomal deposits of Gb3 may function as damage-associated molecular patterns (DAMPs) or induce the production of DAMPs by damaged cells and induce pro-oxidative and pro-apoptotic patterns (4,16,23).

De Francesco et al. (16) reported that unstimulated PBMC from FD patients had higher levels of both IL-1β and TNF-α, whereas IL-6 levels were not different from those of controls. After 24h of cell culture, these authors found the elevated production of IL-1β and IL-6, but not TNF-α. Gb3 was associated with the release of the cytokines IL-1β and TNF-α, but not IL-6, and enhanced the activity of dendritic cells and monocytes. When a TLR4 inhibitor was used, the inflammatory response was blocked, suggesting an interaction between Gb3 and this receptor. The participation of the innate immune system in this response has been highlighted. Our results show elevated serum IL-6 and TNF-α levels in classic FD patients, while the IL-1β levels were unchanged. It is not possible to directly compare cellular expression *in vitro* to the serum levels *in vivo* because cytokines are released in a paracrine manner and serum levels may vary depending on several factors, such as receptor binding, temperature-associated degradation, and breakdown within reacting cells when the subject’´s blood sample is drawn (24).

Recently, Üçeyler et al. (17) found the increased expression of TNF, IL-1β, and TLR4 in PBMCs from men with FD. However, the secreted TNF levels were only increased in PBMCs from men with FD with pain and classical variants and not in those without pain. Nevertheless, it was demonstrated that heat and lipopolysaccharide stimuli enhance TNF secretion and Gb3 accumulation, linking typical triggers with FD pain.

Biancini et al. (14), using a multi-analyte panel similar to that presented herein, evaluated 14 FD patients being treated with ERT and found elevated plasma levels of IL-6 and TNF-α compared to those in controls, and also advocated for the role of Gb3 as a promoter of inflammation. However, the IL-1β levels were not measured in their study. Similarly, we found elevated serum levels of IL-6 and TNF-α, but not IL-1β, in patients with FD. It is noteworthy that both the study by Biancini et al. and our present study included patients being treated with ERT, which may have influenced the observed results. Notably, the elevated levels of IL-6 found in the patients studied herein was largely due to the results from men with FD, but when comparing patients undergoing ERT to those that were not, the elevated IL-6 levels were driven by the women with FD. Sex-associated differences in plasma IL-6 levels are not well understood. The same cut-off value is typically used for both sexes. Pooling of data for men and women with FD may not be accurate, and reference values may require different cut-off values (25). Thus, data from our study were presented as both a pooled analysis and separated based on sex. Overall, our results suggest that patients with more severe disease, and thus, receiving ERT, have higher serum IL-6 and TNF-α levels than untreated patients.

The MSSI score is a marker of disease severity. The original work of Whybra et al. (19) showed that FD patients undergoing ERT showed reduced MSSI scores after one year of treatment with agalsidase alfa, particularly on account of general, neurological, and cardiovascular sub-scores. The present study was a transverse assessment of FD patients at different disease time points. The majority of the patients enrolled in this study were cared for by outside providers; they had different access to healthcare and data acquisition at regular time points was not possible for many patients. Although the results for the 24-hour urine protein or microalbumin measurements and echocardiograms were obtained for most patients, we only collected information on broadly defined parameters, as required to calculate the MSSI scores. Thus, it was not possible to assess the trend of how the MSSI scores changed over time, nor to compare the parameters to calculate the Fabry Stabilization Index (FASTEX), a marker of the clinical stabilization of the disease (26).

**Figure S1:**
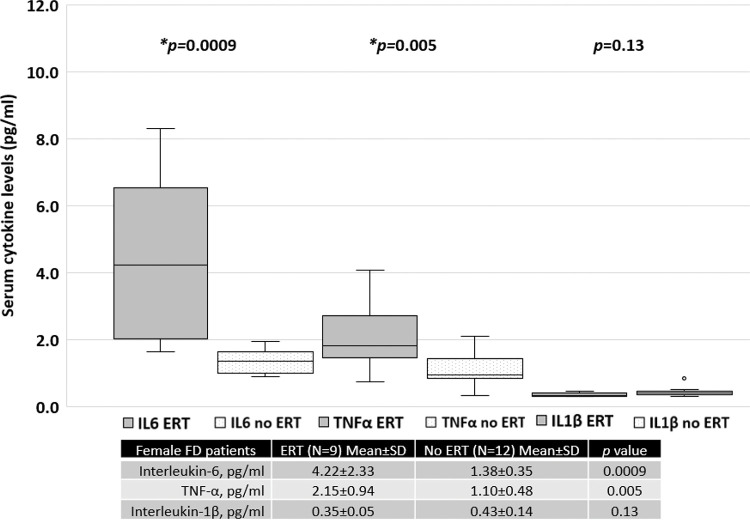
Serum cytokine levels in women with FD treated with ERT *versus* those not treated with ERT.

**Figure S2:**
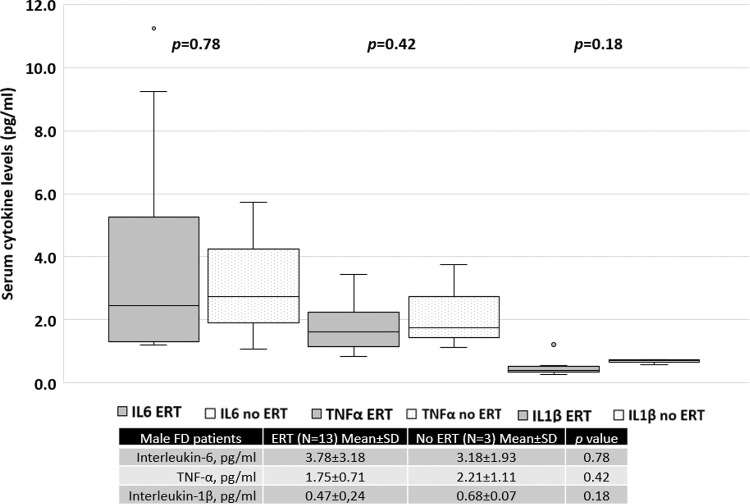
Serum cytokine levels in men with FD treated with ERT *versus* those not treated with ERT.

With regard to only women with FD, those undergoing ERT had higher MSSI scores, which reflects the current treatment guidelines, which state that women with FD who are symptomatic or present with organ dysfunction are more likely to be prescribed specific treatment. Therefore, higher serum cytokine levels in this group may be related to a higher disease burden, illustrating the indication for targeted treatment (Figure S1, refer to Appendix). Moreover, the average MSSI scores and serum cytokine levels in men with FD treated with and without ERT were similar. However, only three men with FD were not being treated with ERT, precluding proper analysis (Figure S2, Appendix).

Interestingly, Safyan et al. (17) studied polymorphisms of key pro- and anti-inflammatory cytokine-encoding genes in FD, namely, *IL-1β*, *IL-1α*, *TNF-α*, and IL-10. These authors found that most patients had low TNF-α levels and high levels of IL-10, IL-1β, and IL-1α. They described a correlation between the MSSI renal and neurological sub-scores and TNF-α levels (*p*=0.06) and the MSSI neurological sub-score and IL-10 levels (*p*=0.03); however, the levels of none of the cytokines were correlated with alpha-galactosidase A levels. Our results indicate the opposite, with patients having elevated serum TNF-α levels and a positive correlation of the levels of this cytokine with higher MSSI scores.

Given that patients being treated with ERT in our study presented with elevated levels of pro-inflammatory cytokines, an observation that has not been consistently reported, this raises several questions: a) whether ERT alone fails to adequately suppress the inflammatory process; b) whether ERT itself contributes to the inflammatory process; c) whether specialists should research the possible use of anti-inflammatory or immunomodulatory drugs in the care of patients with FD; and d) whether there is a point of no return after which ERT is no longer beneficial for a specific target organ and the degree of tissue damage (or fibrosis) may contribute to persistent inflammation and lead to organ failure regardless of the treatment. As reviewed by Rozenfeld and Feriozzi (4), fibrosis is a common finding in the heart and kidney of FD patients; it is driven by TGF-β1 and TLR4 activation. 

This study also has a few limitations. We evaluated the serum levels of these cytokines, while previous publications reported their plasma or PBMC levels; therefore, these values are not directly comparable. As a transverse study, the measurement of cytokine levels was performed only once, and the cytokine levels prior to ERT or at the time of FD diagnosis were not measured. Similarly, the MSSI scores were assessed only once, and it is not possible to evaluate the impact of ERT on these results, to establish a trend of MSSI scores, or to calculate the FASTEX scores. Interpretation of the correlations between the cytokine levels and MSSI scores reported in this study should consider all these aspects. In addition, the low number of untreated men with FD hampered additional comparisons.

Nevertheless, a thorough review was performed to ensure the validity of the MSSI scores at the time of assessment. The patients showed different degrees of kidney and heart damage, and several parameters, including hemodialysis, patient status post kidney transplantation, treatment with different immunosuppressive regimens, delayed time to adequate diagnosis, and specific treatment initiation, were considered in this review. 

## CONCLUSION

In the patient cohort studied herein, FD was associated with elevated serum levels of IL-6 and TNF-α. Patients with FD that received ERT, particularly, women, showed higher levels of serum IL-6 and TNF-α than those that did not receive ERT; the levels of serum IL-6 and TNF-α were correlated with the MSSI scores reflecting a greater disease burden. Further studies assessing the levels of inflammatory cytokines pre- and post-infusion in treatment-naïve patients and comparisons to migalastat-treated patients prior to therapy initiation and after determined timepoints may provide additional information regarding this topic.

### Conflicts of Interests

NSRN declares having received speaker’s and advisory board fees from Shire HGT, now Takeda Pharmaceuticals. JCBB declares that their spouse received speaker’s and advisory board fees from Shire HGT, now Takeda Pharmaceuticals.

## AUTHOR CONTRIBUTIONS

Rosa Neto NS designed the study, collected, analyzed, interpreted the data and wrote the manuscript. Bento JCB collected the data. Caparbo VF collected the data. Pereira RMR analyzed and interpreted the data and reviewed the manuscript. All of the authors have read and approved the final version of the manuscript.
